# Drug discovery for chemotherapeutic resistance based on pathway-responsive gene sets and its application in breast cancer

**DOI:** 10.3389/fbinf.2025.1661601

**Published:** 2025-09-16

**Authors:** Dehua Feng, Jingwen Hao, Lingxu Li, Jian Chen, Xinying Liu, Ruijie Zhang, Huirui Han, Tianyi Li, Xuefeng Wang, Xia Li, Lei Yu, Bing Li, Jin Li, Limei Wang

**Affiliations:** 1 School of Intelligent Medicine and Technology, Kidney Disease Research Institute, Hainan Engineering Research Center for Health Big Data, Hainan Medical University, Haikou, Hainan, China; 2 Key Laboratory of Brain Science Research and Transformation in Tropical Environment of Hainan Province, Department of Anatomy, School of Basic Medicine, Hainan Medical University, Haikou, Hainan, China

**Keywords:** chemotherapeutic resistance, pathway-responsive gene sets, drug discovery, drug combination therapy, precision oncology

## Abstract

**Introduction:**

Chemotherapy response variability in cancer patients necessitates novel strategies targeting chemoresistant populations. While combinatorial regimens show promise through synergistic pharmacological interactions, traditional pathway enrichment methods relying on static gene sets fail to capture drug-induced dynamic transcriptional perturbations.

**Methods:**

To address this challenge, we developed the Pathway-Responsive Gene Sets (PRGS) framework to systematically identify chemoresistance-associated pathways and guide therapeutic intervention. Comparative evaluation of three computational strategies (GSEA-like method, Hypergeometric test-based method, Bates test-based method) revealed that the GSEA-like methodology exhibited superior performance, enabling precise identification of drug-induced pathway dysregulation.

**Results:**

Key experimental findings demonstrated PRGS’s superiority over conventional Pathway Member Gene Sets (PMGS), exhibiting statistical independence (*p* < 0.0001) and enhanced detection of chemotherapy-driven pathway dysregulation. Application of PRGS to the GDSC dataset identified 8 resistance-associated pathways. Screening of agents targeting these pathways yielded candidates with predicted anti-resistance activity. An *in vitro* cellular experiment demonstrated that the bortezomib-bleomycin combination exhibited synergistic cytotoxicity (IDAcomboScore = 0.014) in T47D cells, highlighting the potential of PRGS-guided therapeutic strategies.

**Discussion:**

This study establishes a PRGS-based methodological framework that integrates genomic perturbations with precision oncology, demonstrating its capacity to decode resistance mechanisms and guide therapeutic development through dynamic pathway analysis.

## 1 Introduction

Chemoresistance remains a critical obstacle in cancer treatment, driven by the multifaceted mechanisms through which cancer cells subvert chemotherapeutic efficacy (Talib et al., 2021). Tumor heterogeneity, particularly intra-tumoral genetic and phenotypic diversity, significantly contributes to therapeutic failure by enabling dynamic adaptation to treatment pressures (Fernandez et al., 2018). To overcome this complexity, combinatorial strategies incorporating pathway inhibitors have emerged as a paradigm shift in overcoming drug resistance. These inhibitors specifically target oncogenic signaling cascades to effectively attenuate chemotherapy resistance. They achieve this by simultaneously blocking adaptive survival pathways that cancer cells activate under treatment stress and disrupting pro-survival signaling networks that confer drug tolerance, thereby enhancing therapeutic efficacy (Neel and Bivona, 2017). For instance, Sui et al. (2010) demonstrated that pharmacological inhibition of the estrogen receptor pathway overcomes resistance to vinca alkaloids in breast cancer models, highlighting the potential for targeted co-therapies. Such synergistic interactions between pathway modulators and conventional chemotherapeutics not only enhance treatment efficacy but also provide mechanistic insights into resistance antagonism, paving the way for precision oncology approaches (Song et al., 2022).

Traditional pathway gene annotations are predominantly curated through subjective methodologies, wherein constituent genes exhibit functionally coordinated interactions to execute specific biological processes or pathways (Chicco and Agapito, 2022). These gene sets are compiled from heterogeneous sources, including curated pathway databases (Kanehisa and Goto, 2000), biomedical literature mining (Venet et al., 2011), and domain expert knowledge systems (Zhang et al., 2011). Classification criteria for pathway membership gene sets (PMGS) vary widely, ranging from genes co-participating in canonical signaling cascades to those sharing homologous functional annotations (Li et al., 2017). Notably, when biological systems are perturbed by pharmacological agents, a subset of genes demonstrating robust transcriptional reprogramming, termed “response signatures”, emerges as critical biomarkers for deciphering context-specific therapeutic mechanisms (Atkinson et al., 2019). This conceptual divergence between PMGS and response signatures lies in their foundational definitions: the former is pre-defined through static pathway frameworks, while the latter arises dynamically from observed transcriptional adaptations to pathological stimuli. Such mechanistic dichotomy underscores the necessity for a dynamically informed model that integrates pathway response signatures into existing pathway frameworks. This approach further leverages treatment-perturbed pharmacodynamic profiles to refine therapeutic target prioritization, effectively decoupling analysis from static gene set definitions.

A substantial body of publicly available perturbation experiments has been systematically curated to identify treatment-responsive genes (Rydenfelt et al., 2020). These studies employ differential expression profiling to compare perturbed and control conditions, enabling the detection of genes exhibiting consistent regulatory responses to specific interventions (Parikh et al., 2010). Functionally, these genes are characterized by their tight association with perturbed biological pathways. This collective set is defined as pathway-responsive gene sets (PRGS).

Emerging evidence underscores the critical association between post-treatment response gene signatures and therapeutic outcomes, including both drug sensitivity and acquired resistance. Functional interrogation of these genes has revealed their dual capacity to delineate pathway perturbations driving resistance while simultaneously identifying actionable therapeutic targets. For instance, Cai et al. (2023) demonstrated in breast cancer models that palbociclib (a CDK4/6 inhibitor) induced significant upregulation of cyclin-dependent kinase regulators (CDK4, Cyclin D1, Cyclin E1), which mechanistically activated the PI3K/AKT/mTOR axis through a feedback loop involving Cyclin D1 overexpression. Notably, co-administration of PI3K/mTOR/AKT inhibitors disrupted this circuit by reducing Cyclin D1 levels, effectively attenuating palbociclib resistance. Similarly, Zhao et al. (2024) elucidated a gefitinib resistance mechanism in non-small cell lung cancer (NSCLC) driven by S6K1 hyperactivation, a process mediated through the ELK1/mTOR/S6K1 signaling axis. Pharmacological inhibition of S6K1 using the selective inhibitor PF-4708671 not only attenuated gefitinib resistance in resistant models, while simultaneously enhancing anti-tumor efficacy through targeted pathway suppression, thereby establishing S6K1 as a master regulator of adaptive resistance.

In SPEED (Parikh, Klinger, Xia, Marto and Bluthgen, 2010) and SPEED2 (Rydenfelt, Klinger, Klunemann and Bluthgen, 2020), numerous experimental perturbation datasets have been utilized. These datasets could be employed to construct the PRGS, which maps treatment-induced gene expression to drug sensitivity/resistance profiles, uncovering the mechanistic relationship between pathway dysregulation and therapeutic outcomes.

The evolution of enrichment analysis methodologies has been driven by the need to address limitations in detecting biologically meaningful gene sets. Over-representation analysis (ORA), a foundational method in enrichment analysis, employs the Hypergeometric test to assess statistical over-representation of differentially expressed genes (DEGs) within predefined pathways (Liu et al., 2022). While computationally efficient, ORA exhibits inherent sensitivity biases, particularly failing to capture genes with subtle expression changes that contribute meaningfully to biological processes (Peng et al., 2023). To overcome these constraints, Gene Set Enrichment Analysis (GSEA) introduced a paradigm shift by evaluating pathway enrichment through Kolmogorov-Smirnov statistics applied to ranked gene lists, prioritizing collective pathway behavior over individual gene metrics (Cai et al., 2022; Hou et al., 2020). Concurrently, the Bates test expanded the analytical framework by modeling gene set mean rank shifts through Bates distribution approximations, enabling robust detection of moderate but biologically relevant pathway dysregulation (Rydenfelt, Klinger, Klunemann and Bluthgen, 2020). This tripartite methodological framework, ORA for hypothesis-driven single-gene prioritization, GSEA for systems-level pathway interrogation, and the Bates test for quantitative ranking analysis, was systematically applied to PRGS, achieving synergistic validation of pathway-dysregulation hypotheses through complementary analytical perspectives.

In this study, we aim to identify potential therapeutic agents to address chemoresistance in cancer treatment. We constructed PRGS by systematically integrating multiple experimental perturbation datasets. Subsequent application of enrichment analysis methods (GSEA-like, Hypergeometric test-based, Bates test-based) to PRGS in GDSC data revealed candidate drugs with the capacity to reduce cancer cell resistance. Finally, experimental validation was conducted to assess the synergistic therapeutic effects of these drug combinations.

## 2 Materials and methods

**FIGURE 1:**
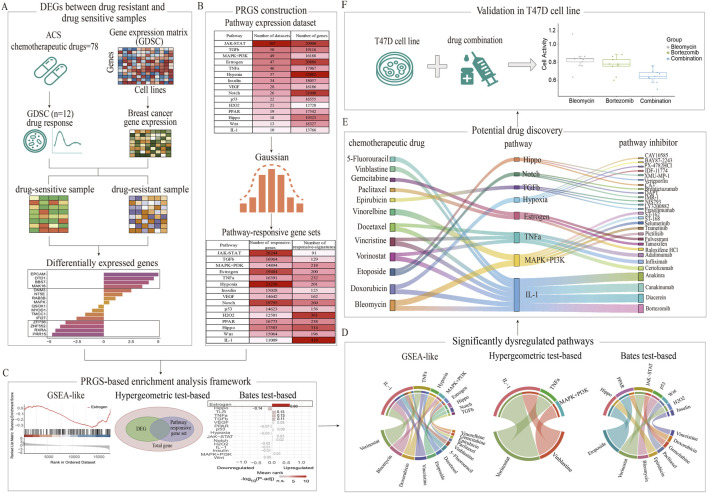
Comprehensive workflow for identifying chemoresistance-associated pathways and therapeutic candidates through PRGS-based enrichment analysis frameworks. **(A)** DEGs were systematically identified between drug-sensitive and drug-resistant groups using GDSC data. **(B)** PRGS was constructed by integrating multiple experimental perturbation datasets. **(C)** A comparative analysis of three PRGS-based enrichment methods (GSEA-like, Bates test-based, and Hypergeometric test-based) was performed to identify pathways associated with chemotherapy resistance. **(D)** Significant pathways linked to chemoresistance were identified between drug-resistant and drug-sensitive groups. **(E)** Potential therapeutic candidates capable of mitigating chemoresistance were discovered based on these pathways. **(F)** The synergistic effect of the bortezomib-bleomycin combination was experimentally validated in T47D cells.

The overall process of data preparation, differential expression analysis, dataset construction, enrichment analysis, and cellular experimental validation in this study is shown in the flowchart (Figure 1).

### 2.1 Data sources

#### 2.1.1 Pathway perturbation-response gene expression datasets

The experimental perturbation-response datasets were obtained from the study previously published by Rydenfelt et al. (2020), containing 15 pathways, 534 experimental datasets, and 29,133 genes. Each pathway contains multiple experimental datasets.

#### 2.1.2 Drug sensitivity data in breast cancer cell line

We curated a panel of 78 chemotherapeutic agents approved by the American Cancer Society (ACS, https://www.cancer.org/) (Narayanan and Honavar, 2017). Chemosensitivity profiles were retrieved from the Genomics of Drug Sensitivity in Cancer (GDSC) database for breast cancer cell lines, with complete datasets available for 19 agents (Yang et al., 2013). To ensure statistical robustness, compounds exhibiting extreme response bias (defined as >90% of cell lines showing uniform resistance or sensitivity) were excluded, eliminating seven agents. This selection yielded 12 drugs with balanced resistance/sensitivity distributions for quantitative analysis: 5-fluorouracil; bleomycin; docetaxel; doxorubicin; epirubicin; etoposide; gemcitabine; paclitaxel; vinblastine; vincristine; vinorelbine; and vorinostat.

The GDSC repository provided chemosensitivity profiles across 51 breast cancer cell lines, with individual drug sensitivity data coverage ranging from 38 to 51 cell lines per agent among the 12 selected agents. Drug sensitivity was quantified using the area under the concentration-response curve (AUC) metric. Drug-sensitive and drug-resistant groups were defined using predefined AUC thresholds: drug-sensitive groups by AUC<0.8 and drug-resistant groups by AUC≥0.8 (Callari et al., 2023). The sensitivity of each chemotherapeutic agent was ranked in descending order based on AUC values. Subsequently, drug-sensitive and drug-resistant groups were identified through threshold-based selection, prioritizing the lowest eight sensitive and highest 8–12 resistant cell lines.

#### 2.1.3 Gene expression in breast cancer cell line

Gene expression profiling data comprising 17,737 genes across 1,018 cancer cell lines were retrieved from the GDSC database. Subsequently, gene expression data were extracted from breast cancer cell lines for drug sensitivity analysis, encompassing 17,419 genes and 51 distinct breast cancer cell lines.

### 2.2 Differentially expressed genes between drug-sensitive and drug-resistant groups

Breast cancer datasets were categorized into drug-sensitive and drug-resistant groups based on responses to 12 chemotherapeutic agents (5-fluorouracil, paclitaxel, *etc.*). DEGs analysis was conducted using independent samples t-tests, with statistical significance defined as *p*-value < 0.01.

### 2.3 Pathway-responsive gene sets construction

For each experimental perturbation dataset, genes with expression below the median were excluded. When a gene was expressed in multiple datasets, the z-value corresponding to the highest expression was selected; conversely, for genes expressed in a single dataset, their observed z-values were retained. An adjusted z-value reflecting gene expression changes specific to each perturbation type was calculated using Formula 1:

$$z_{adjusted}=z_{raw}*sign\left(type\right)$$ (1)

where z_adjusted_ represents the adjusted z-value for the gene, z_raw_ represents the raw z-value for gene expression, the sign represents the signum function (returning 1 for input > 0, 0 for input = 0, or −1 for input<0), and type represents the perturbation types (assigning 1 for activation and −1 for inhibition).

For each pathway, z-transformed values were derived by applying Gaussian transformation to all adjusted z-values within each dataset (Hanzelmann et al., 2013) (Supplementary Figure S1).

Subsequently, the mean of these z-transformed values across all experiments was calculated for each gene (hereafter termed z-score), with genes expressed in fewer than two datasets excluded from the analysis.

Pathway-related gene signatures (PRGS) were identified using a stringent statistical threshold |z-score|>2.58, *p*-value < 0.01).

### 2.4 PRGS-based enrichment analysis

We conducted systematic pathway enrichment analysis using the PRGS framework to identify dysregulated pathways between chemoresistant and chemosensitive breast cancer subtypes. Pathway significance was determined with *p*-values (*p* < 0.01). Three methodologies were utilized in the analysis: GSEA-like, Bates test-based, and Hypergeometric test-based analyses, each implemented with distinct input variable configurations to identify relevant pathways. Within the PRGS framework, variables were categorized into continuous and discrete types based on their characteristics and roles in pathway analysis. Continuous PRGS were defined as genes within pathways that underwent computational processing without statistical thresholding, retaining quantitative measurements of gene expression changes. Discrete PRGS were defined as signature genes meeting stringent statistical criteria and closely associated with pathway regulation. Total genes were derived from the gene expression dataset following independent t-test analyses, retaining their processed were obtained by applying statistical thresholds to these total genes, identifying those significantly associated with drug sensitivity or resistance.

**FIGURE 2:**
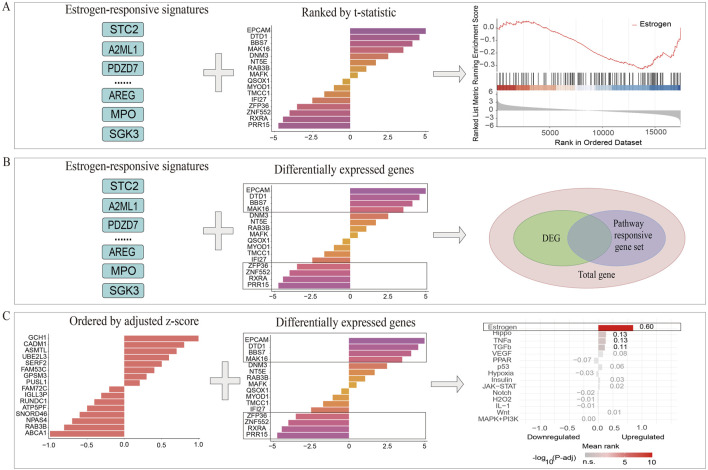
Systematic workflow for PRGS-based enrichment analysis methodologies. **(A)** In the GSEA-like methodology, genes are ranked using t-statistics, and PRGS enrichment is assessed within the generated ranked gene list. **(B)** The overlap between DEGs and PRGS is evaluated by the Hypergeometric test-based method, and a *p*-value is calculated to determine the significance of pathway enrichment. **(C)** Within the Bates test-based method, genes are ranked using adjusted z-scores, and PRGS enrichment is evaluated within the resulting ranked list.

Input variables for the three PRGS-based enrichment analysis methods were as follows: discrete PRGS and total genes were used for the GSEA-like methodology; continuous PRGS and DEGs were employed for the Bates test-based methodology; DEGs and discrete PRGS were used for the Hypergeometric test-based methodology (Figure 2).

#### 2.4.1 GSEA-like methodology

In GSEA-like method (Figure 2A), genes were ranked based on t-statistics, and the enrichment score (ES) for each pathway was calculated. The distribution of the ES for the *j*th gene g in the *i*th position of PRGS was calculated using a modified Kolmogorov-Smirnov statistic. The ES was evaluated by calculating the maximum absolute deviation between P_hit_ and P_miss_, as defined by Formula 2–4 (Marczyk et al., 2021):

$$P_{\text{hit}}\left(S,i\right)=\sum_{\begin{array}{c} g_{j}\in S\\ j\leq i \end{array}}\frac{{\left|r_{j}\right|}^{p}}{N_{R}}where\;N_{R}=\sum_{g_{j}\in S}{\left|r_{j}\right|}^{p}$$ (2)

Where S represents the PRGS, r is the ranked score, *p* is a weighting parameter, N is the number of total genes in the ranked list, and N_R_ is calculated by summing the ranks of genes within PRGS.

$$P_{miss}\left(S,i\right)=\sum_{\begin{array}{c} g_{j}\notin S\\ j\leq i \end{array}}\frac{1}{\left(N-N_{H}\right)}$$ (3)

Where N_H_ is the number of genes in the PRGS.

$$ES=P_{\text{hit}}-P_{\text{miss}}$$ (4)

Where P_hit_ represents the weighted sum of genes within PRGS, P_miss_ represents the weighted sum of genes outside PRGS. The ES increases when a gene is part of a gene set and decreases otherwise. This process was repeated for PRGS and total genes in each group.

#### 2.4.2 Hypergeometric test-based methodology

The Hypergeometric test-based method was employed to ascertain the statistical significance of a given number of successes (k) in a sample size (n) observed within a larger total size (N), under the assumption that the total population encompasses a total of (M) occurrences of the specific characteristic being analyzed (Elia Venanzi et al., 2024). In this study, PRGS-based Hypergeometric test-based enrichment analysis was conducted on DEGs to identify significant over-expression within the PRGS (Figure 2B). The probability of observing at least k overlapping genes between DEGs and PRGS was calculated by Formula 5:

$$P\left(X\geq k\right)=1-\sum_{i=0}^{k‐1}\frac{\left(\begin{array}{c} M\\ i \end{array}\right)*\left(\begin{array}{c} N-M\\ n-i \end{array}\right)}{\left(\begin{array}{c} N\\ n \end{array}\right)}$$ (5)

Where N represents the total number of genes, n is the number of DEGs, M is the number of overlapping genes between total genes and PRGS, and k is the number of overlapping genes between DEGs and PRGS. A smaller *p*-value indicates a higher likelihood of DEG enrichment within the PRGS.

#### 2.4.3 Bates test-based methodology

The significance of pathway rankings under the null hypothesis was evaluated through the Bates test-based methodology (Figure 2C), which employs a null distribution modeled as the arithmetic mean of N independent uniform variables spanning the interval [−1, 1]. For each biological pathway, differential expression z-values were computed as the ratio of experimental fold changes (stimulated condition vs. control condition) to LOESS regression-derived standard deviations, effectively normalizing expression variation across experimental datasets. These raw z-values underwent Gaussian transformation to address distributional skewness, yielding z-transformed metrics (z-transformed value) with improved normality characteristics. Subsequent analytical steps included: (i) calculation of pathway mean z-transformed values across all N experiments, (ii) Quantile normalization to mitigate batch effects, and (iii) iterative sorting-rescaling procedures that transformed normalized means into final z-scores bounded within [−1,1]. Gene ranking within each PRGS was ultimately determined by these standardized z-scores, as defined in Formula 6:

$$r\left(g\right)=\left\{\begin{array}{c} z_{m}+1e-50;\;if\;z_{m}\left(g\right)\geq \frac{U_{a}+U_{b}}{2}\\ z_{m}-1e-50;\;if\;z_{m}\left(g\right)<\frac{U_{a}+U_{b}}{2} \end{array}\right.$$ (6)

Where z_m_ is the mean z-value of gene, U_a_ = 1 and U_b_ = −1 represent the upper and lower bounds of the standardized interval. The second rank was performed using Formula 7, scaling the initial rank to the interval [−1,1].

$$r^{\prime }\left(g\right)=\left\{\begin{array}{c} -1-r\left(g\right);\;if\;r\left(g\right)\leq 0\\ 1-r\left(g\right);\;if\;r\left(g\right)>0 \end{array}\right.$$ (7)

The r'(g) values of all genes are sorted in ascending order, and each gene is assigned a unique rank from 1 to n based on its position, resulting in the third rank r''(g). Subsequently, these r''(g) values are scaled to the interval [−1,1], yielding the fourth rank r'''(g). Within the context of a designated set of DEGs, overlapping genes were pinpointed through the intersection of PRGS and DEGs. Subsequently, the corresponding r'''(g) values for these overlapping genes were retrieved. The mean rank R(G) was computed by averaging these r'''(g) values, as delineated in Formula 8:

$$R\left(G\right)=\frac{\sum_{i=1}^{n}r^{\prime \prime \prime }\left(g\right)}{n}$$ (8)

Where n is the number of overlapping genes, r'''(g) is the value of the fourth rank. The Bates *p*-value was calculated based on the mean rank and the number of overlapping genes using Formula 9:

$$P\left(x\right)=\left\{\begin{array}{l} batesCDF\left(x,n\right);\;if\;x\leq \frac{a+b}{2}\\ 1-batesCDF\left(\frac{a+b-x}{2},n\right);\;if\;x>\frac{a+b}{2} \end{array}\right.$$ (9)

Where x is the R(G), n is the number of overlapping genes, a corresponds to U_a_ and is assigned a value of 1, while b corresponds to U_b_ and is assigned a value of −1.

### 2.5 Discovery of therapeutic inhibitors

Based on the statistical significance determined by PRGS-based analysis, the top 1-2 pathways most strongly associated with chemoresistance were prioritized as therapeutic targets for each chemotherapeutic agent. Targeted inhibitors were identified by screening the Selleck platform (Wojtaszek et al., 2019) (https://www.selleck.cn/index.html) and PubMed, adhering to the following criteria: (i) inhibitors that directly target pathways identified by PRGS; (ii) among pathways with multiple targeted inhibitors, priority should be given to drugs that have been validated through *in vitro* and *in vivo* experiments, employed in numerous studies and demonstrate broad applicability; (iii) drugs that modulate specific pathways indirectly were also considered; (iv) relevant inhibitors were sourced from PubMed-indexed preclinical research; (v) extracts and plant-derived compounds were excluded.

### 2.6 *In vitro* validation

The predicted synergistic interaction between bortezomib and bleomycin was systematically validated through a two-phase pharmacodynamic evaluation in STR-authenticated T47D breast cancer cells maintained under standardized culture conditions (37 °C, 5% CO_2_). In the first phase, single-agent dose-response profiling was performed to determine the optimal concentrations for subsequent combination experiments. Bortezomib (0.5–40 nM) and bleomycin (50–400 nM) were tested in eight technical replicates per concentration. Dose-response curves revealed that 200 nM bleomycin corresponded to the optimal IC50 threshold concentration, establishing it as the reference concentration for combination studies. In the second phase, combination experiments were conducted by exposing cells to a fixed concentration of 200 nM bleomycin combined with a gradient of bortezomib (0.5–40 nM). Each treatment arm was performed in eight technical replicates to ensure robustness of the data. After 48 h of co-culture, cell viability was quantified using the Cell Counting Kit-8 (CCK-8) *via* absorbance measurement at 450 nm. Synergistic efficacy was quantitatively assessed using the IDAcomboScore method as described by Ling and Huang (2020). Full details of all experimental protocols are available in Supplementary Material.

## 3 Results

### 3.1 Pathway-responsive gene sets

Heterogeneous data distribution across cross-experimental gene expression datasets poses significant challenges to robust analytical outcomes, particularly when dealing with heavy-tailed distributions that violate normality assumptions. To address this issue, we systematically evaluated three normalization strategies to determine the most suitable preprocessing approach.

The most appropriate method was selected from Gaussian normalization, Logarithmic transformation, and Quantile normalization. Using the GSE13837 dataset as an illustrative example, 70% of the data points were initially distributed within the interval [−20, 20], while the remaining 30% exhibited a heavy-tailed distribution outside this interval (Supplementary Figure S2). After normalization, Gaussian normalization markedly diminished distributional heterogeneity, concentrating 90% of the data points within the narrower interval of [−3, 3], while the ratio of negative to positive values was balanced, changing from 58.9:41.1 to 49.6:50.4 (Supplementary Figure S3).

In contrast, the Logarithmic transformation resulted in data clustering predominantly within the range [−5, 5], while the overall distribution remained skewed. Notably, the ratio of negative to positive values shifted markedly to 25.1:74.9.

Although the distribution resulting from Quantile normalization approximates a normal distribution within the interval [−15, 15], a limited number of extreme values persist beyond these boundaries. Similar patterns were observed in the other two datasets (Supplementary Figures S2 and S3).

Overall, Gaussian normalization emerged as the optimal method. It maps data onto a standard normal distribution, effectively compressing extreme values and improving distributional balance.

Substantial inter-dataset distributional disparities were observed, and we systematically applied Gaussian normalization to all pathway-derived datasets, achieving scale consistency across expression datasets (Hai et al., 2023).

A systematic analysis of these post-normalization datasets spanning multiple pathways identified genes with consistent regulation patterns across experimental conditions, enabling the construction of PRGS (Supplementary Figure S4A). Differences in the number of datasets across pathways were observed, with IL-1 (9 datasets) and Wnt (12 datasets) having relatively small numbers of experimental data, while JAK-STAT (66 datasets) and TGFa (44 datasets) had relatively large numbers. This variation may stem from differences in experimental design, the diversity of available perturbation reagents, or the maturity of research on different pathways. Quantitative comparison revealed that the IL-1 pathway exhibited the highest number of responsive signatures (410), which may be attributed to its role as a key pro-inflammatory factor that triggers extensive transcriptional changes (Supplementary Figure S4B). These changes are associated with numerous biological processes, including immune responses, cell proliferation, and apoptosis, where significant gene expression alterations occur (Xiao et al., 2022). In contrast, the JAK-STAT pathway exhibited the fewest responsive genes, with only 91 identified. This may indicate that fewer genes meet the criteria for consistent response under the experimental conditions used.

Despite variations in the number of responsive genes, they provide a robust foundation for subsequent functional analysis, enabling more effective identification of pathway dysregulation in specific biological samples. For instance, in a study by Gregory et al., their analysis of dysregulated genes in response to imatinib therapy identified the Wnt pathway as a therapeutic target (Gregory et al., 2010).

### 3.2 Differential expression of drug-sensitive genes across drug groups

DEGs between drug-sensitive and resistant breast cancer cell groups across 12 chemotherapeutic agents were identified through differential expression analysis. To focus on genes with significant directional expression changes, we applied an additional filter (|t-statistic|>2.8) to prioritize biologically impactful candidates. This analysis revealed substantial heterogeneity in t-statistic distributions across agents (Table 1; Supplementary Figure S5A), indicating that chemotherapy resistance-associated pathway dysregulation exhibits distinct molecular signatures under different drug regimens.

**TABLE 1:** The number of DEGs of drug-sensitive genes.

Drug	Number of upregulated genes	Number of downregulated genes	Total number of DEGs	Total sample size
Paclitaxel	218	256	474	21
Gemcitabine	108	162	270	21
Etoposide	148	103	251	21
Bleomycin	113	120	233	21
Vorinostat	95	101	196	20
Vinblastine	110	82	192	20
Vincristine	82	74	156	22
5-Fluorouracil	130	19	149	20
Vinorelbine	65	43	108	20
Doxorubicin	55	41	96	21
Epirubicin	40	47	87	21
Docetaxel	38	21	59	21

In cancer, downregulated tumorous suppressor genes have been linked to chemotherapy resistance (Tallen et al., 2003). Half of the DEGs in the some analyzed group showed a tendency toward downregulation (i.e., a greater number of downregulated genes than upregulated genes). Conversely, the remaining DEGs of sample groups exhibited an opposite expression pattern, suggesting potential differences in their chemoresistance mechanisms. Additionally, significant expression changes of some DEGs were only observed in the paclitaxel- or gemcitabine-treatment group, with minimal overlap between these groups (Supplementary Figure S5B). These findings indicated a potential association between these genes and resistance to specific pharmaceutical agents.

### 3.3 Evaluation of different enrichment methods

**FIGURE 3:**
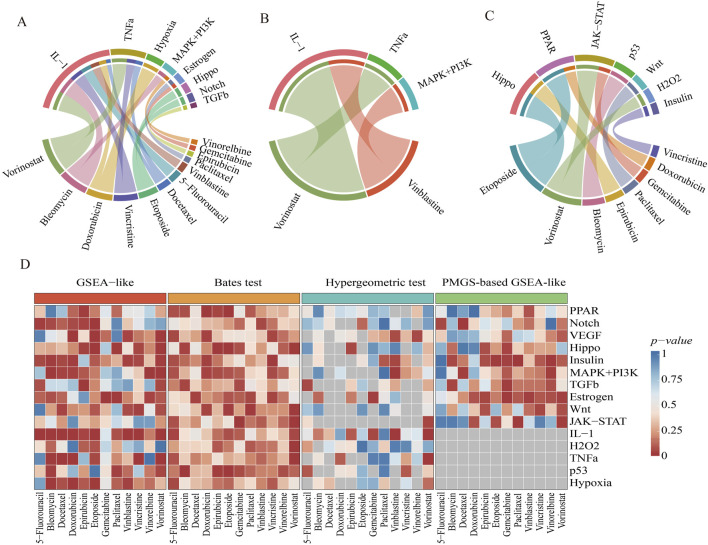
Identification of significant pathways by three enrichment analyses. **(A–C)** Significant pathways were identified using three enrichment analysis methods: GSEA-like, Hypergeometric test-based, and Bates test-based. A chord diagram illustrates the complex relationships between pathways and drug-sensitive/resistant groups, with the width of each band reflecting the strength of these relationships. **(D)** A comparison of enrichment results across different drug-sensitive/resistant groups is shown. The x-axis represents drug-sensitive/resistant samples, while the y-axis represents 15 pathways. Color intensity indicates the significance of enrichment *p*-values, with grey indicating pathways not identified by the method. This visualization highlights the performance differences among the three methodologies.

Three enrichment analysis methodologies were systematically applied to the PRGS framework using drug response-associated DEGs. These genes were analyzed to compare drug-sensitive and -resistant subgroups within the GDSC dataset, facilitating the identification of pathways driving chemoresistance. The GSEA-like methodology demonstrated superior performance, identifying eight key pathways (*p*-value < 0.01) with strong activation of IL-1 and TNFα pathways in multidrug-resistant groups (Figure 3A). Notably, both of which were inflammatory pathways. Functional interrogation further uncovered shared regulatory nodes (e.g., NF-kB activation) between these two pathways, mechanistically linking their coactivation to sustained chemoresistant phenotypes (Feng et al., 2025; Gao et al., 2024).

Three chemoresistance-associated pathways were identified through Hypergeometric test-based methodology across two drug-sensitive/resistant subgroup comparisons (Figure 3B). The statistical significance of IL-1 and TNFα pathways was consistently validated by both Hypergeometric test-based and GSEA-like methodologies. Gene-sharing limitations between PRGS and DEGs resulted in undetectable key pathways for 5-fluorouracil, bleomycin, and docetaxel groups (Figure 3D).

Seven chemoresistance-associated pathways were identified through Bates test-based analysis, with Hippo, PPAR, and JAK-STAT pathways showing consistent activation across multiple subgroups (Figure 3C). Functional validation confirmed these pathways as master regulators of chemoresistance acquisition in preclinical models (Nascimento et al., 2017; Zeng and Dong, 2021; Zhang et al., 2024). The statistical comparison revealed that the Bates test-based methodology identified significantly fewer chemoresistance-associated pathways compared to the GSEA-like methodology (Figure 3D).

The comparative analysis demonstrated that pathways identified through the GSEA-like methodology achieved superior statistical significance (*p* < 0.0001). Significant overlap was observed between pathways identified by the GSEA-like methodology and those detected by alternative approaches, with the GSEA-like methodology identifying the highest number of pathways across all evaluated methods. These findings supported the conclusion that the GSEA-like methodology represents the optimal approach for pathway identification.

### 3.4 Comparing differences between PRGS and PMGS

Gene sets for ten pathways were retrieved from the KEGG database to evaluate differences between PRGS and PMGS. The Jaccard index and the number of overlaps for each pathway were calculated (Supplementary Figure S6; Supplementary Table S1). The results show extremely low overlap between PRGS and PMGS across all pathways, with an average Jaccard index of only 0.012 (range: 0–0.036).

From a biological perspective, this limited overlap stems from their distinct design objectives. PMGS includes genes that are annotated as components of well-known canonical pathways and signaling cascades (Gerling et al., 2013). These encompass pathway receptors, kinases, transcription factors and co-regulators whose functions remain unaffected by external perturbations (Rashidiani et al., 2024). To illustrate this point, consider the estrogen pathway. In estrogen receptor-positive (ER+) breast cancers that HER2 and EGFR are overexpressed, downstream signaling components can be activated. This activation subsequently enhances the activity of the estrogen receptor and its co-activator AIB1, thereby facilitating the estrogen agonistic activity of tamoxifen in breast cancer (Rahem et al., 2020). This phenomenon results from dysregulated pathway activity due to aberrant gene function within the PMGS. In contrast, PRGS comprises genes whose expression levels are markedly upregulated or downregulated in response to external perturbations affecting the pathway (Wang, Karikomi, et al., 2019). Within the estrogen pathway, 17β-estradiol (E2) acts as a perturbing ligand that binds to estrogen receptor α (ERα) in the cytoplasm. This interaction induces a conformational change in ERα, prompting its translocation to the nucleus, where it modulates gene transcription by binding to estrogen response elements (EREs) located in the promoter regions of target genes (Krolick and Shi, 2022). These target genes, which constitute the PRGS, are not core pathway components but rather represent downstream “response products” elicited following pathway activation.

The GSEA-like methodology demonstrated superior performance when applied to PRGS using GDSC data (*p* < 0.0001). Subsequent implementation of the GSEA-like approach on PMGS identified four key pathways (Supplementary Figure S7). The Insulin and JAK-STAT pathways have been previously reported to be associated with chemoresistance (Han et al., 2021; Macaulay et al., 2013). The GSEA-like methodology is based on differential expression rankings, which are inherently supported by the continuous gene expression values of PRGS. In contrast, PMGS-based analysis produced statistically significant but less robust enrichment outcomes (*p <* 0.01), as its pathway annotations lack quantitative expression data required for optimal GSEA-like implementation (Figure 3D). This discrepancy arises because PRGS captures dynamic pathway regulation through expression magnitude, while PMGS only reflects static gene set membership.

### 3.5 Discovering potential drugs to overcome chemoresistance in breast cancer

**FIGURE 4:**
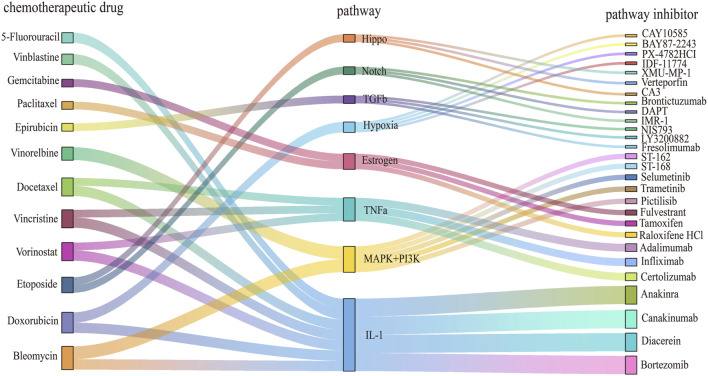
Discovery of drug candidates to overcome chemoresistance in cancer treatment. The Sankey diagram illustrates the relationships between chemotherapeutic drugs, dysregulated pathways, and pathway inhibitors. Nodes on the left represent chemotherapeutic agents for cancer treatment, central nodes correspond to dysregulated pathways associated with these drugs, and nodes on the right indicate pathway inhibitors capable of modulating these pathways. Distinct colors and node shapes correspond to various chemotherapy agents, dysregulated pathways, and inhibitors, respectively. The connecting lines illustrate the directional flow between these three elements.

Eight key pathways associated with chemoresistance in breast cancer were identified using the PRGS-based GSEA-like approach. These pathways are dysregulated in response to specific chemotherapeutic agents, and can be targeted for therapeutic intervention with pathway-specific inhibitors (Figure 4). In line with the screening criteria, the selected pathway inhibitors were obtained from the Selleck platform and relevant literature indexed in PubMed.

The estrogen pathway was found to be dysregulated in paclitaxel-resistant subgroups. Tamoxifen and fulvestrant, both explicitly classified as “estrogen receptor antagonists” in the Selleck database, function by inhibiting estrogen activity and suppressing the proliferation of breast cancer cells. Previous studies have shown that fulvestrant can make estrogen receptor-negative breast tumors more susceptible to chemotherapy, whereas tamoxifen can mitigate paclitaxel resistance mediated by ERα-positive signaling (Jiang et al., 2014; Nzegwu et al., 2021). Although both clomifene citrate and tamoxifen are estrogen receptor modulators, clomifene citrate has limited applicability and lacks sufficient experimental validation in cancer models. In contrast, tamoxifen is supported by Phase II clinical trial data and exhibits greater practical utility, with multiple *in vitro* studies confirming its capacity to induce apoptosis in breast cancer cells. Consequently, tamoxifen and fulvestrant are viable inhibitors for the direct targeting of the estrogen pathway.

Bortezomib, while primarily a proteasome inhibitor, also inhibits NF-kB activity. Its combined administration with IL-1 receptor antagonists has been shown to reduce tumor formation and growth *in vivo* (McLoed et al., 2016). In patients with rheumatoid arthritis, bortezomib suppresses the release of pro-inflammatory cytokines TNFα and IL-1β (Maseda et al., 2014). Therefore, it serves as an indirect inhibitor of the IL-1 pathway.

In addition, drugs that had previously been validated as inhibitors of specific pathways were considered. ST-162 and ST-168 are small-molecule bifunctional inhibitors targeting both the MEK and PI3K pathways, demonstrating dose-dependent suppression of MEK and PI3K signal transduction (Smith et al., 2018). They are being developed as novel anti-tumor agents, but are not currently documented in the Selleck platform. Thus, they represent potential inhibitors for targeting the MAPK+ PI3K pathways.

Promising drug combinations targeting resistant cell lines were identified through this workflow (Supplementary Table S2). A notable example is the HCC1428 paclitaxel-resistant cells (AUC = 0.95), exhibited significant estrogen pathway dysregulation (Yang et al., 2013). Fulvestrant was determined to be an inhibitor of the estrogen pathway (Wakeling et al., 1991). Therefore, they were considered as a potential therapeutic combination. These findings provide diverse combination therapy strategies to overcome chemoresistance in breast cancer.

### 3.6 Bleomycin-bortezomib synergistic effects validation in T47D cells

Both IL-1 and TNFα pathways were identified as key pathways in both drug-sensitive and drug-resistant subgroups through GSEA-like and Hypergeometric test-based analyses.

Studies have shown that the IL-1 pathway increases drug resistance by activating and reinforcing the release of pro-inflammatory cytokines (Stanam et al., 2016). Moreover, in breast cancer cells exhibiting high sensitivity to IL-1β, stimulation with IL-1β markedly induces the upregulation of BIRC3 expression, consequently conferring resistance to doxorubicin treatment (Rho et al., 2022). In alignment with these findings, BIRC3 is identified as a responsive gene within the IL-1-responsive gene set, thereby strongly suggesting its role as a mediator of IL-1-induced chemoresistance.

Overexpression of TNFα can fragment tumor DNA, thereby contributing to resistance to the cytotoxic effects of chemotherapeutic agents (Adrian et al., 2023). TNFα can also activate the NF-kB signalling pathway and promote the expression of CXCL1/2, which leads to the amplification of the CXCL1/2-S100A8/9 loop and induces chemotherapy resistance (Cruceriu et al., 2020). Consistently, the presence of CXCL1 and CXCL2 genes associated with the TNFα-responsive gene set was observed, suggesting that this mechanism likely plays a significant role in TNFα-mediated drug resistance.

The T47D cell line represents the luminal A subtype of breast cancer, characterized by positivity for estrogen receptor (ER+) and progesterone receptor (PR+) and human epidermal growth factor receptor 2 (HER2-) (Ahmed et al., 2022). This subtype constitutes approximately 70% of all breast cancer cases (Fiorillo et al., 2020). The proliferation of T47D cell was inhibited upon exposure to both native and recombinant human interleukin-1 (IL-1) isoforms, specifically the α and β variants (Gaffney and Tsai, 1986). Importantly, analysis using PRGS has identified the IL-1 pathway as a key therapeutic target in the bleomycin response subgroup. Bortezomib exhibits anti-inflammatory activity and suppresses IL-1 (Mao et al., 2012). These findings suggest the potential for synergistic benefits in combining bleomycin with IL-1 inhibitors for use in patients who do not respond to bleomycin. Consequently, bortezomib was selected for combination treatment with bleomycin in T47D cell.

**FIGURE 5:**
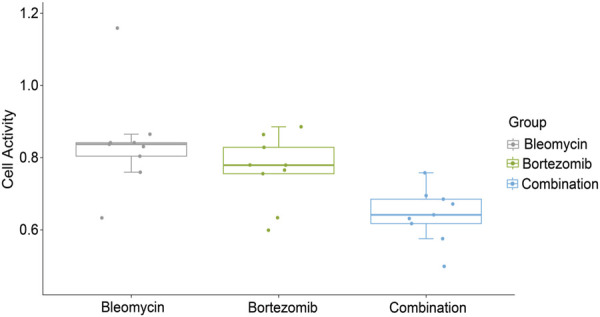
The effect of bleomycin, bortezomib monotherapy and their combination on T47D cell viability. The x-axis represents the different treatment groups (bleomycin, bortezomib, combination therapy), while the y-axis represents cell viability.

Under optimal drug concentrations, this combination reduced cellular viability to 20% (Figure 5), with bleomycin and bortezomib demonstrating IC50 values of 200 nmol/L and 40 nmol/L, respectively. An IDAcomboscore of 0.014 (threshold≥0.004 for synergy) confirmed significant interaction between bleomycin and bortezomib.

## 4 Discussion

Despite the increasing application of gene set analysis, systematic investigations into gene set selection criteria remain limited. The impact of distinct gene set definitions on analytical outcomes remains underexplored. Two gene set categories, PRGS and PMGS, were compared in this study. PMGS comprise curated gene sets associated with biological processes, chromosomal locations, disease associations, or pathway membership (Hermida et al., 2013), reflecting their functional annotation purposes. Conversely, PRGS capture pathway-specific expression changes under perturbation conditions, maintaining causal relationships between gene expression and pathway activity. Significant differences in gene composition were observed between PMGS and PRGS, with limited overlap indicating divergent design objectives. While PRGS genes may overlap with PMGS, reciprocal inclusion was not observed. Consequently, PRGS were prioritized to characterize chemotherapy resistance-associated expression patterns.

Three enrichment analysis methodologies based on PRGS were implemented. The GSEA-like approach demonstrated superior performance, identifying a greater number of statistically significant pathways compared to alternative methods. This superiority was attributed to its emphasis on aggregate gene set trends within ranked lists. Notably, predefined gene lists exhibited substantial differential expression levels, contributing to substantial pathway enrichment. The Bates test-based method involves multiple sorting of gene z-values, focusing on the average ranking of genes within a given gene set. Conversely, Hypergeometric test-based analysis was constrained by limited gene overlap between PRGS and DEGs, with overlapping gene quantity prioritized over expression magnitude. This methodological limitation resulted in the lowest number of detected pathways among all evaluated approaches.

Current strategies to address chemotherapeutic resistance include combination therapies, nanomedicine platforms, and gene therapeutic approaches. A self-delivery nanomedicine incorporating α-tocopherol succinate and doxorubicin was developed by Zheng et al. to overcome drug resistance through synergistic chemotherapy (Zheng et al., 2021). Similarly, CRISPR/Cas9-mediated gene editing was employed to disrupt uPAR expression, resulting in enhanced chemosensitivity of HCT 8/T cells (Wang et al., 2019). In this study, pathway inhibitors were combined with chemotherapeutic agents to target dysregulated pathways underlying chemoresistance. The methodology proposed herein exhibits broad applicability beyond chemotherapy resistance, extending to endocrine drug and other drug class-associated resistance mechanisms through combination with pathway inhibitors. As an illustrative example, Dong et al. demonstrated that PI3K/AKT/mTOR pathway inhibition restores estrogen receptor signaling, thereby overcoming resistance to endocrine treatments in preclinical models (Dong et al., 2021). Drug combination of bleomycin and bortezomib was validated in T47D cell line. However, more experiments need to be performed in the following studies.

In summary, this study highlights the importance of PRGS in characterizing chemotherapy resistance-associated expression patterns. By prioritizing PRGS over PMGS, we were able to capture dynamic pathway-specific expression changes and their causal relationships with pathway activity, providing a more accurate representation of biological mechanisms underlying chemoresistance. The GSEA-like approach, with its focus on aggregate gene set trends, outperformed other methodologies in identifying statistically significant pathways, underscoring its utility in pathway analysis. These findings not only advance our understanding of chemoresistance mechanisms but also offer a robust framework for developing targeted therapeutic strategies. The proposed method exhibits broad applicability beyond chemotherapy resistance, extending to other drug class-associated resistance mechanisms through the integration of pathway inhibitors. Thus it provides a foundation for future research aimed at overcoming treatment resistance and improving therapeutic outcomes in cancer patients.

## Data Availability

The original contributions presented in the study are included in the article/Supplementary Material, further inquiries can be directed to the corresponding authors.
