# Supplementary material for: Drug discovery for chemotherapeutic resistance based on pathway-responsive gene sets and its application in breast cancer
Source: Front Bioinform. 2025 Sep 16;5:1661601. doi: 10.3389/fbinf.2025.1661601 (PMC12479470; doi:10.3389/fbinf.2025.1661601)
Supplement: Supplementary file 1 [file Supplementaryfile1.docx]

Supplementary Material

# Supplementary Methods

1.1 Reagents and apparatus

Carbon dioxide incubator, biological safety cabinet, low-speed centrifuge, micropipette and tips, 1.5 mL Eppendorf tubes,15 mL and 50 mL centrifuge tubes, dimethyl sulfoxide (DMSO; cell culture grade; Selleck D8371), 1640 medium (Gibco), T47D cells were purchased from the Pricella (authenticated using STR profile analysis), T47D cells specific medium (Pricella, CM-0228), Cell Counting Kit-8 (CCK-8) Cell Proliferation and Cytotoxicity Assay Kit (CA1210, Solarbio), count cells used hemacytometer, sterile PBS buffer, cells were digested with 0.25% trypsin solution (Bi Yun Tian Biotechnological Co.Ltd, Shanghai, China), cell culture bottles, 96-well cell culture plates, Bleomycin (NSC12506, Selleck), Bortezomib (S1013, Selleck).

1.2 Cell subculture and growth conditions

T47D cells were used within two weeks of receipt and authenticated by short tandem repeat (STR) profiling. Cells were cultured in tissue flasks and passaged at 70%-85% confluence. After incubation, the culture medium was aspirated, and the residual culture solution was washed with 1 ml of phosphate-buffered saline (PBS). The PBS was discarded, and 1mL of trypsin-ethylene diamine tetra acetic acid (EDTA) solution was added for digestion at 37 degrees C for 2 minutes. After that, 1mL of T47D cell-specific medium was added to terminate the digestion. The cells in the culture flask were blown down to prepare a cell suspension. The cell suspension was transferred to a 15 mL centrifuge tube and was centrifuged at 1200g for 2 minutes at room temperature. After centrifugation, the supernatant was removed and a fresh medium was added. The culture medium was refreshed every 48 hours, and cells were sub-cultured at a ratio of 1:3.

1.3 Cell processing and culture

Fresh medium was added to prepare the cell suspension and the cells were counted. A single-cell suspension was obtained by diluting the suspension to 50,000 cells/ml and then culturing. A cell suspension containing 10,000 cells (200 µL) was added to each well in a 96-well plate. Subsequently, the plates were incubated for 24 hours at 37 degrees C in a 5% carbon dioxide (CO2) humidified atmosphere in a cell culture incubator.

1.4 Determination of optimal concentrations for combination therapy

The drug concentrations were selected based on effective doses determined in previous studies (Shankavaram et al. 2009, Thaler et al. 2015, Yang et al. 2013). Bortezomib was used in concentrations ranging from 2.5 to 40 nM, while the NCI-60 panel employed three bleomycin concentrations (10 nM, 100 nM, and 1,000 nM) for T47D cell treatment. Within the GDSC, T47D cells were exposed to bleomycin concentrations spanning from 250 nM to 64,000 nM. The specific concentration ranges for the two drugs in combination therapy were determined through subsequent measurements. For single-drug experiments, cells were treated with six bortezomib concentrations (0.5, 2.5, 10, 20, 30, and 40 nmol/L) and four bleomycin concentrations (50, 100, 200, and 400 nmol/L), with each concentration tested in eight replicates. The aim was to select an appropriate concentration range to evaluate cellular sensitivity to the drugs. The optimal concentration of bleomycin was determined to be 200 nmol/L. Bortezomib at various concentrations was combined with 200 nmol/L bleomycin, and the effects on cell activity were observed. Cell viability was assessed after a 48-hour exposure to ascertain the initial concentrations, defined as the optimal concentrations selected based on the impact of varying drug concentrations on cell growth inhibition rates. In combination therapy experiments, cells were exposed to both drugs together, with eight replicates per treatment group.

1.5 Cell viability measurement by CCK-8 method

The drug-containing culture medium was removed and replaced with a fresh culture medium containing 100 μL of the drug. After drug treatment, 10 μL of CCK-8 reagent was added to each well, followed by incubation for 1 hour at 37 degrees C in 5% CO2 atmosphere with 95% humidity. Cell viability was assessed by measuring the optical density (OD) at 450 nm using a microplate reader. The viability percentage was calculated using the formula: cell viability (%)=[A (dosing) / A (not dosing)] * 100%. Where A (doing) represented the OD value of the well with cells, CCK-8 solution, and drug solution. A (not dosing) represented the OD value of the well with cells and CCK-8 solution but no drug solution.

# Supplementary Figures and Tables

## Supplementary Figures

**
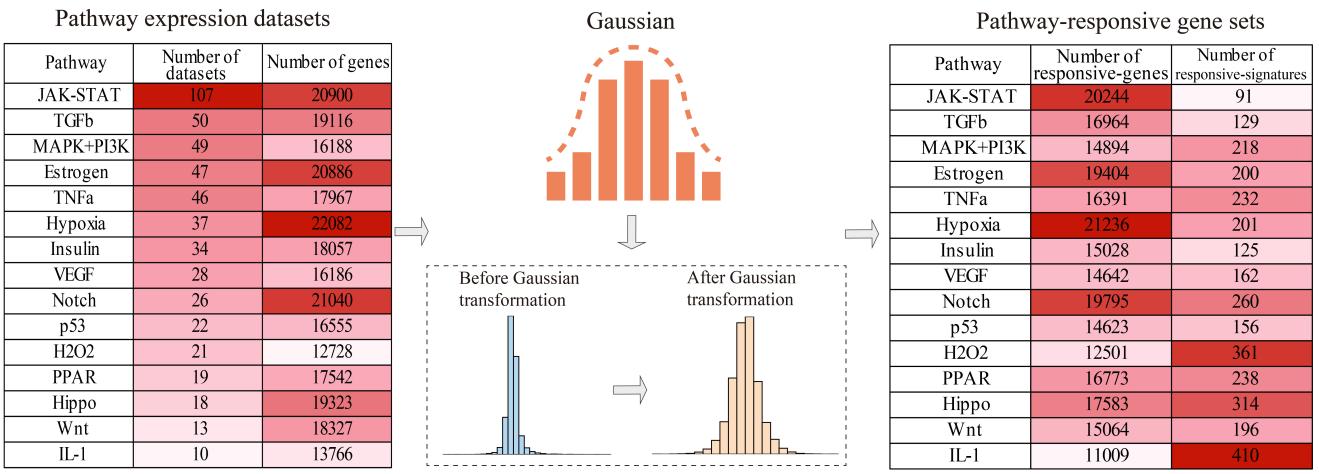
**

**Supplementary Figure 1.** The construction of PRGS. From left to right, it details the number of experimental datasets and genes for each pathway, the Gaussian transformation process applied to each dataset, and the resulting number of genes and responsive signatures for each pathway.





**Supplementary Figure 2.** A comparison of the distributions of gene expression values across three datasets before and after processing. The columns represent three gene expression datasets, including GSE13837 (left), GSE16650 (middle) and GSE22807 (right). The rows show the data distributions for Pre-processing, Gaussian transformation, Log transformation and Quantile normalization. In each distribution plot, the x-axis represents the range of gene expression values, while the y-axis shows the frequency.


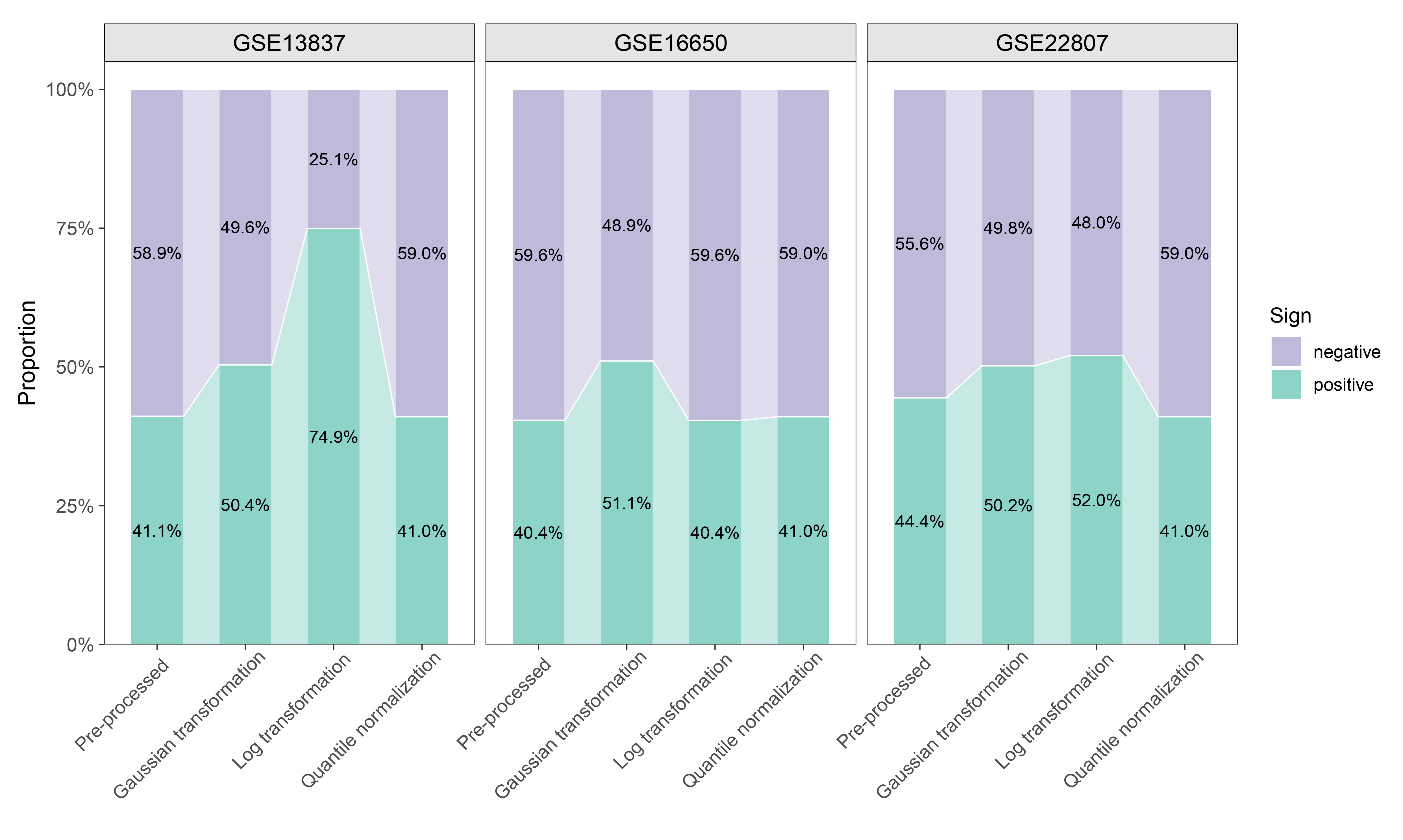


**Supplementary Figure 3.** Proportion of negative and positive gene expression values across three datasets, both before and after processing. The columns correspond to the GSE13837, GSE16650 and GSE22807 datasets. The rows illustrate the pre-processed, Gaussian transformation, Log transformation and Quantile normalization. The y-axis shows the proportion of values, with purple denoting negative values and green denoting positive values.


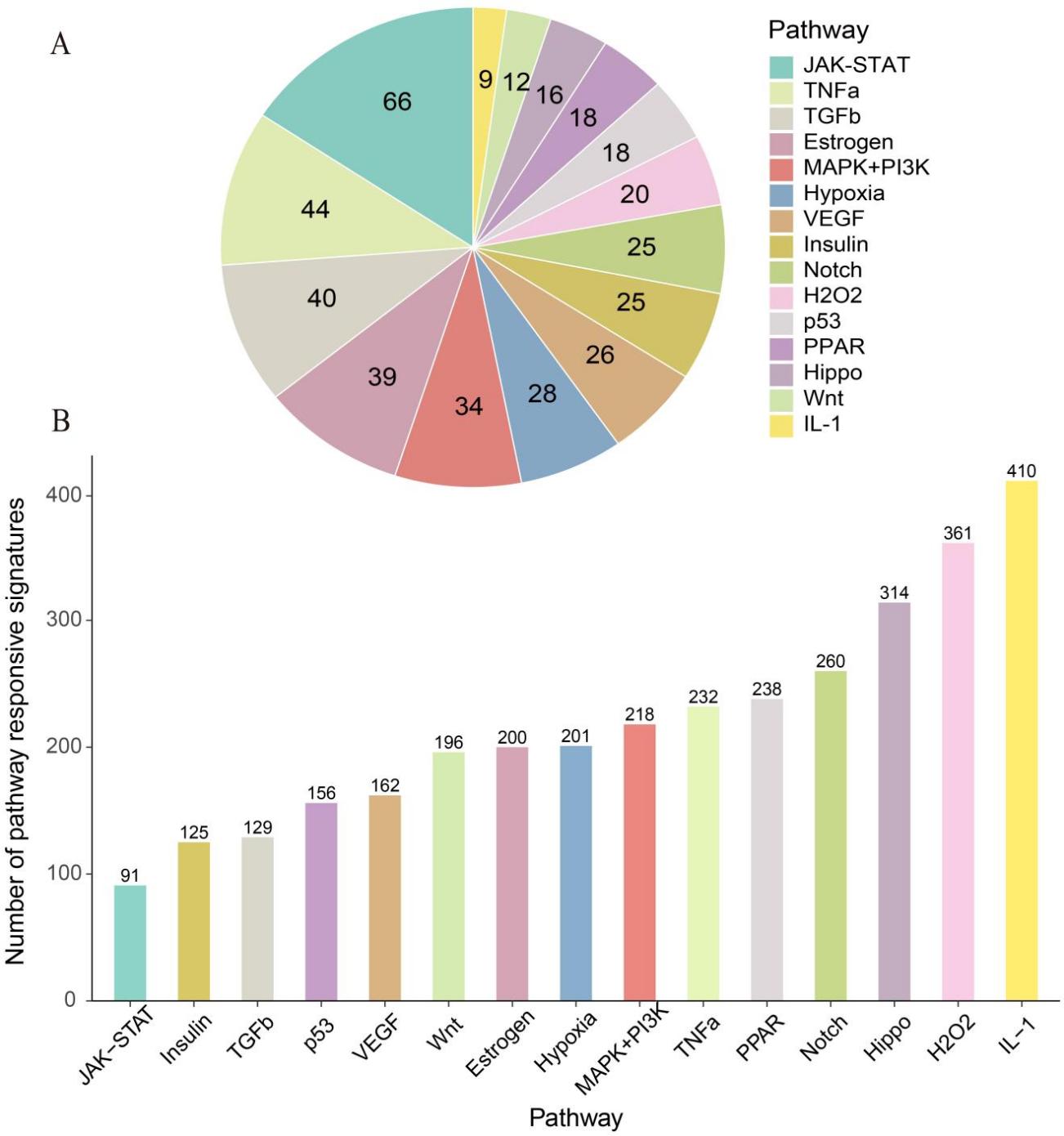


**Supplementary Figure 4.** Overview of the number of experimental datasets and responsive signatures in PRGS. **(A)** Distribution of the number of experimental datasets across different pathway categories in PRGS. **(B)** The x-axis represents the categories of pathways, and the y-axis represents the number of responsive signatures , with different colors indicating distinct pathway categories.


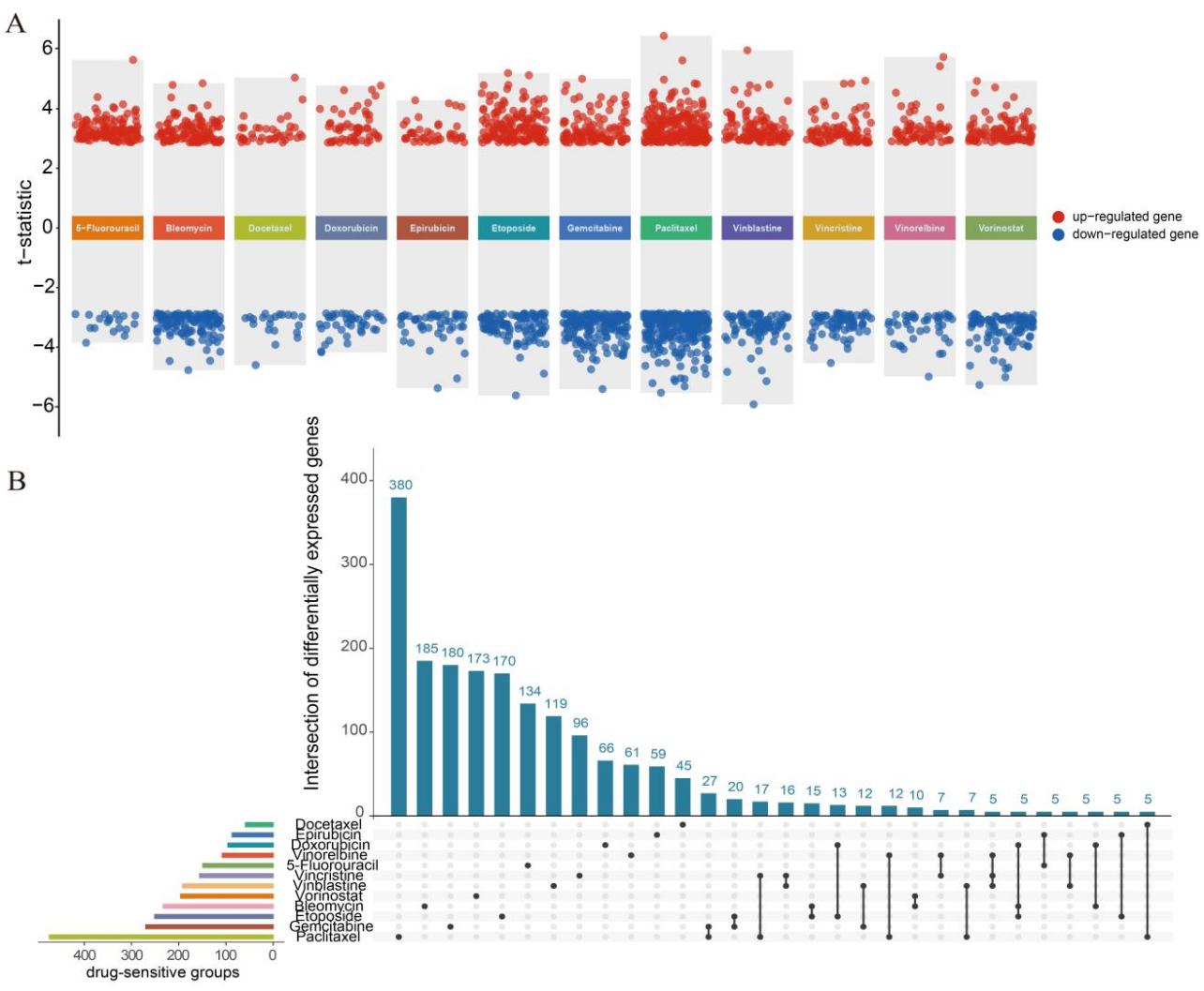


**Supplementary Figure 5.** Comprehensive analysis of DEGs in different drug-sensitive/resistant groups. **(A)** The distribution of t-statistics of DEGs in each drug-sensitive/resistant group is illustrated, indicating the magnitude and direction of gene expression changes. DEGs were identified using a threshold of |t-statistic|>2.8. **(B)** The UpSet plot visualizes the total number of DEGs, the number of specific genes for each drug group, and the overlap of DEGs across different drug groups.


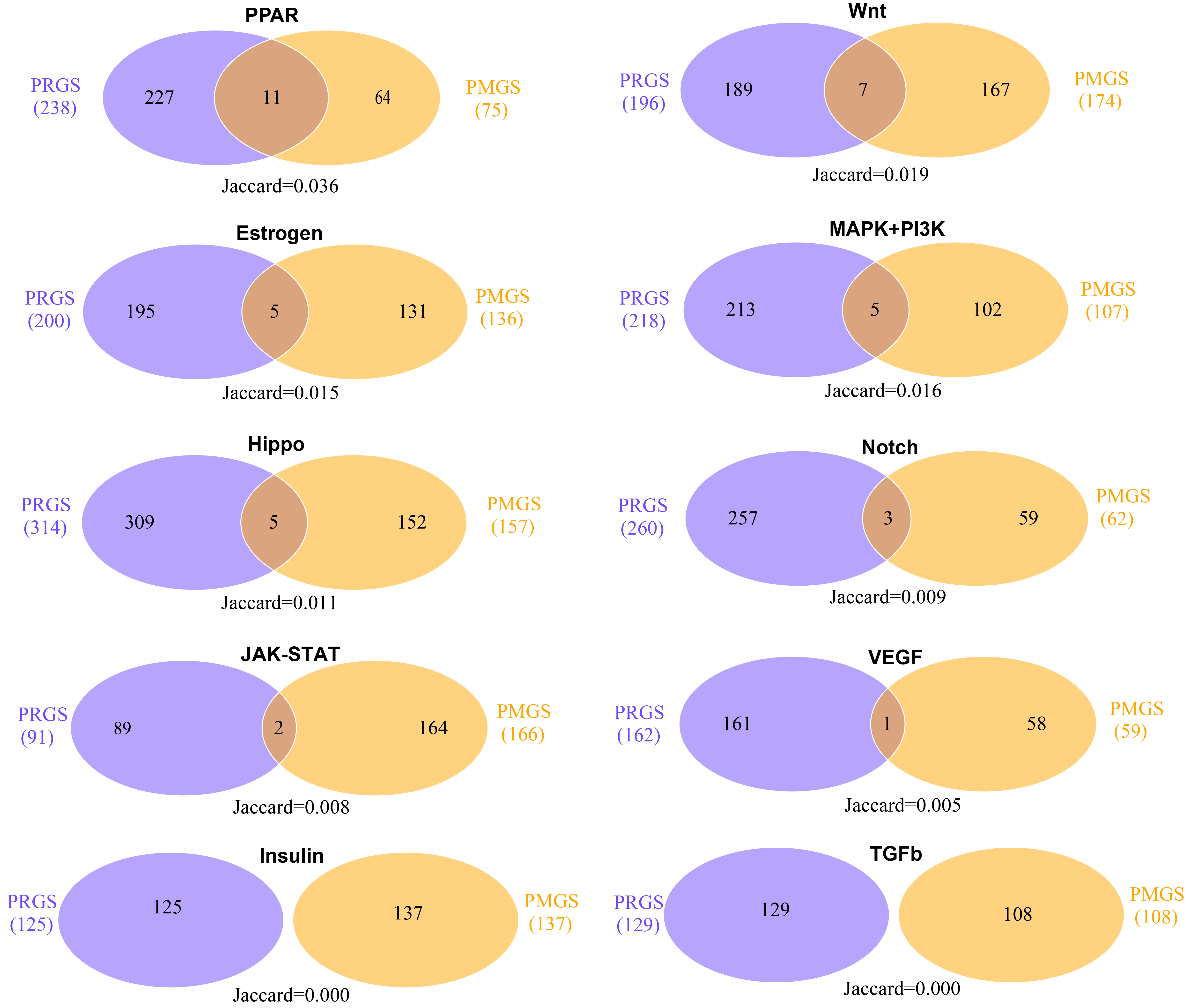


**Supplementary Figure 6.** Gene overlap between PRGS and PMGS across 10 pathways. The purple and yellow circles show the number of genes in PRGS and PMGS respectively, and the overlapping regions show the shared genes. The Jaccard index ranges from 0 to 0.036 and quantifies gene overlap across all pathways.


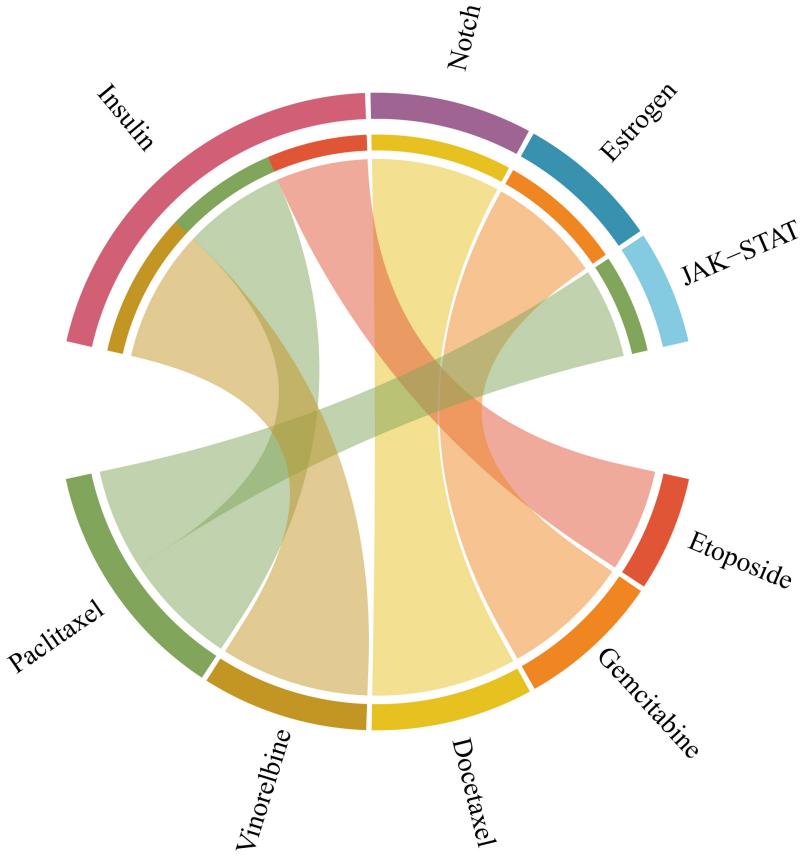


**Supplementary Figure 7.** Significant pathways identified by PMGS-based GSEA-like methodology. The complex relationships between pathways and drug-sensitive/resistant groups were plotted in the chord diagram.

## Supplementary Tables

Supplementary Table 1 The common genes between PMGS and PRGS.

| Pathway | Number of pathway member genes | Number of pathway-responsive signatures | Number of common genes | Jaccard |
| --- | --- | --- | --- | --- |
| PPAR | 75 | 238 | 11 | 0.036 |
| Wnt | 174 | 196 | 7 | 0.019 |
| Estrogen | 136 | 200 | 5 | 0.015 |
| MAPK+PI3K | 107 | 218 | 5 | 0.016 |
| Hippo | 157 | 314 | 5 | 0.011 |
| Notch | 62 | 260 | 3 | 0.009 |
| JAK-STAT | 166 | 91 | 2 | 0.008 |
| VEGF | 59 | 162 | 1 | 0.005 |
| Insulin | 137 | 125 | 0 | 0.000 |
| TGFb | 108 | 129 | 0 | 0.000 |

Supplementary Table 2 Combinations of chemotherapeutic drugs and pathway inhibitors in resistant cell lines.

| Drug-resistant cell lines | Chemotherapeutic drug | Pathway  inhibitor | Pathway | Literature |
| --- | --- | --- | --- | --- |
| HCC1428 | Paclitaxel | Fulvestrant | Estrogen | Fulvestrant, a selective estrogen receptor down-regulator, sensitizes estrogen receptor negative breast tumors to chemotherap |
| BT-483 | Bleomycin | Anakinra | IL-1 | Interleukin-1 and Nuclear Factor Kappa B Signaling Promote Breast Cancer Progression and Treatment Resistance |
| T47D | Bleomycin | Bortezomib | IL-1 | The proteasome inhibitor Bortezomib (Velcade) as potential inhibitor of estrogen receptor-positive breast cancer |
| T47D | Bleomycin | Diacerein | IL-1 | Diacerein-mediated inhibition of IL-6/IL-6R signaling induces apoptotic effects on breast cancer |
| BT-483 | Bleomycin | Canakinumab | IL-1 | Key Factor Regulating Inflammatory Microenvironment, Metastasis, and Resistance in Breast Cancer: Interleukin-1 Signalin |
| BT-483 | Bleomycin | ST-162 | MAPK+PI3K | A bifunctional MAPK/PI3K antagonist for inhibition of tumor growth and metastasis |
| BT-483 | Bleomycin | ST-168 | MAPK+PI3K | Ocular Toxicity Profile of ST-162 and ST-168 as Novel Bifunctional MEK/PI3K Inhibitors |
| HCC1419 | Vinorelbine | ST-162 | MAPK+PI3K | A bifunctional MAPK/PI3K antagonist for inhibition of tumor growth and metastasis |
| HCC1419 | Vinorelbine | ST-168 | MAPK+PI3K | Ocular Toxicity Profile of ST-162 and ST-168 as Novel Bifunctional MEK/PI3K Inhibitors |

References

Shankavaram UT, Varma S, Kane D, Sunshine M, Chary KK, Reinhold WC, Pommier Y, Weinstein JN. 2009. CellMiner: a relational database and query tool for the NCI-60 cancer cell lines. BMC Genomics. Jun 23;10:277. Epub 2009/06/25.

Thaler S, Thiede G, Hengstler JG, Schad A, Schmidt M, Sleeman JP. 2015. The proteasome inhibitor Bortezomib (Velcade) as potential inhibitor of estrogen receptor-positive breast cancer. Int J Cancer. Aug 1;137:686-697. Epub 2014/12/23.

Yang W, Soares J, Greninger P, Edelman EJ, Lightfoot H, Forbes S, Bindal N, Beare D, Smith JA, Thompson IR, et al. 2013. Genomics of Drug Sensitivity in Cancer (GDSC): a resource for therapeutic biomarker discovery in cancer cells. Nucleic Acids Res. Jan;41:D955-961. Epub 2012/11/28.
